# Corruption and Solid Waste Management in Mbarara Municipality, Uganda

**DOI:** 10.1155/2020/4754780

**Published:** 2020-06-29

**Authors:** Pius Gumisiriza, Sylvester Kugonza

**Affiliations:** School of Civil Service, Public Administration and Governance, Uganda Management Institute, P.O. Box 20131, Kampala, Uganda

## Abstract

Mbarara Municipality in Western Uganda has for many years struggled to manage municipal solid waste. Leaders in this municipality have mainly attributed this persistent problem to poor financing, failure to enforce existing solid waste management laws and regulations, limited community participation, deprived attitude by the public towards waste collection, and tendency of municipal dwellers to litter. No in-depth academic study in Mbarara Municipality has ever been done to expose and illustrate how corruption directly happens and influence solid waste management. This study fills this knowledge gap by illustrating how corruption influences poor solid waste management in Mbarara Municipality. The study finds that municipal technical officials, garbage truck drivers, their turn boys, garbage sorters, factory owners, and private land grabbers all involved in different forms of corruption have directly and indirectly turned solid waste collection and disposal into a very costly problem to the municipal council and the general public. The article recommends that fighting corruption in all its forms without fear or favor, encouraging them to play their role particularly in sorting waste, adoption of smart technologies, and putting in place measures that attract private investors while protecting the public can help in the effective management of solid waste in Mbarara Municipality.

## 1. Introduction

Increased municipal solid waste production is a natural consequence of urbanisation, economic, and population growth [1]. Unlike cities/towns in developed countries which have effective mechanism for managing municipal solid waste, those in developing countries face huge challenges [2–6]. Indeed, in Uganda, municipal solid waste is very huge challenge in almost all the cities/municipalities [7]. Most studies and media reports in Uganda have attributed the problem to poor financing, failure to enforce existing waste management laws, political interferences, lack of community participation, poor attitude by the public towards waste collection, inadequate planning, and people's tendency to throw garbage anywhere including environmentally sensitive areas [8–13]. Studies/media in other parts of the world have also indicated that corruption can be a huge hindrance to effective management of municipal solid waste [14–16]. Katusiimeh et al. [17] in their study on the effectiveness of public and private provision of solid waste collection services in Kampala also revealed that city officials use solid waste as a cash cow to milk public money for their own benefits. In Mbarara municipality, angry residents have for years protested about corruption and sordid solid waste management challenges [18–23]. However, no academic study has been undertaken to understand the nature and magnitude of corruption embedded within the solid waste management processes in this municipality a gap filled by this research article.

## 2. Mbarara Municipality and Solid Waste Problems

Mbarara Municipality is located in Mbarara district, southwestern Uganda, 266 kilometers from Kampala the capital city. It has been growing very fast in the last two decades and is currently the biggest town in the western region. The municipality has a total land area of about 51.47 sq. kms and had a total population of 195,160 as of the 2014 national population census. It is made up of three divisions of Kakoba, Kamukuzi, and Nyamitanga [24]. In 2005, Mbarara Municipality became one of the beneficiaries of a US$ 300,000 National Environmental Management Authority–World Bank Solid Waste Composting Plant Project meant to convert organic waste into compost manure [25]. According to the terms of this project, all the municipal solid waste generated in Mbarara Municipality was supposed to be dumped at the 114-acre Kenkombe land in Rwentondo Cell, Kakoba Ward. This land owned by the council is located approximately seven kilometers from Mbarara town Center. The Municipal Council was supposed to provide the overall project implementation and management functions including placement of skips on specified garbage collection points, mobilization of households, and community based and local nongovernmental organisations to ensure effective waste sorting by its generators and proper disposal into the skips. The council was also mandated to contract commendable private companies to carry out waste collection and delivery to the solid waste composting plant at Rwentondo for processing and production, as well as marketing of the produced manure. For project sustainability purposes, communities were supposed to actively participate in on-site sorting of waste, play a key role in monitoring house-to-house waste collection, and pay five hundred shillings (500/=) per month for the service. The market assessment and analysis conducted revealed that there was already a market for the compost manure if it was reasonably priced [26].

**Figure 1:**
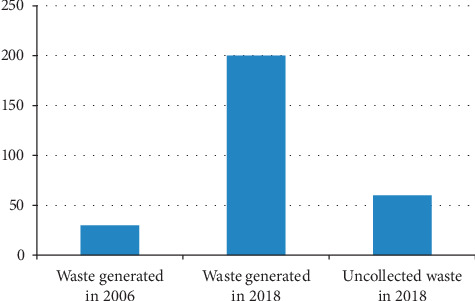
Waste generated and uncollected/collected.

Despite the above comprehensive plans, angry residents of Rwemigyina and Kenkombe villages that border this open garbage-dumping site started complaining about the stinking garbage, aborted foetuses, and huge volumes of leachate that found its way in the nearby Rwentondo stream as early as 2006 and the problem continued [27, 28]. In 2005, the town was generating and collecting around 30 tons of solid waste per day [26]. By the end of 2018, the amount of solid waste generated had skyrocketed to over 200 tons per day which is a very significant increase [9]. Many written reports have revealed that over 30% of the over 200 tons of waste generated in Mbarara Municipality per day remains uncollected, unprocessed, or dumped in inappropriate places [19, 21, 22]. As illustrated by the graph below, it is observable that there have been very sharp increases in the amount of waste generated. It is also clear that the amount of solid waste that remains uncollected per day increased very significantly in the last 10–13 years. In fact, it is double the amount of the solid waste that was generated in 2006 (Figure 1).

**Figure 2:**
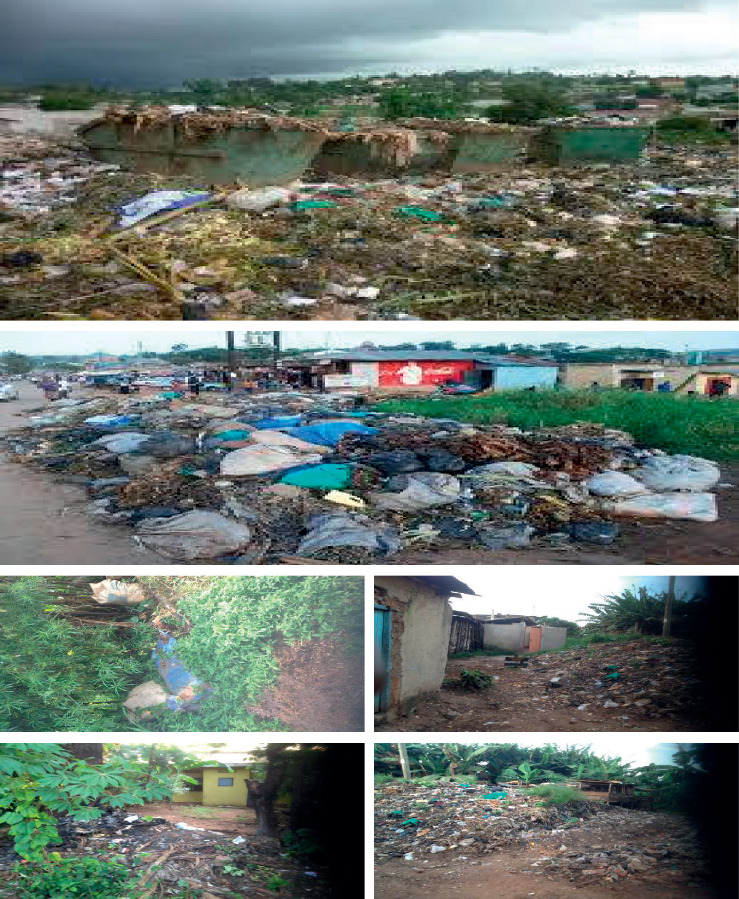
Uncollected waste in different parts of Mbarara Municipality.

These claims are exemplified by a lot of photographic evidence taken by the researchers during data collection for this study and pictures taken by other reporters on the problem as illustrated in Figure 2.

Angry residents of Mbarara have for years complained about environmental, sanitation, and health problems as a consequence of mismanaged municipal solid waste [18–23, 29]. Their complaints are indeed with merit as many other studies have clearly documented that poorly managed solid waste comes with serious consequences including environmental degradation (soil, surface, and ground water pollution), respiratory problems, and human diseases, such as cholera and diarrhea, harms animals that consume waste unknowingly, and affects the poor most [1, 30–33]. This article exposes the nature and influence of corruption embedded in the Mbarara municipality waste management process with an overall aim of reducing corruption and properly managing solid waste in this town and others in Uganda.

## 3. Material and Methods

### 3.1. Data Sources

This article is built on both secondary and primary qualitative data obtained from various credible sources. Secondary data was obtained from Mbarara Municipal Council reports (obtained with permission) and academic and newspaper articles as reflected in the references. Qualitative primary data was collected from 38 purposively sampled key informants who in one way or another have been involved in or affected by solid waste management issues in Mbarara municipality, as detailed in Table 1.

### 3.2. Data Collection Techniques

Corruption is a very sensitive and complex practice which manifests in many ways in different environments [34, 35]. Many respondents that may be involved or know people involved in corruption may not easily or freely give accurate information on the problem. To overcome this challenge, the researchers used different techniques to collect accurate data. First, the questions posed to some respondents particularly garbage truck drivers, turn boys, sorter, and farmers did not directly refer to their practices as corrupt. They were mainly asked about how they sort garbage, where they transport it, how much money is involved per ton, and how it is paid and shared. Similarly farmers were asked about where they buy the compostable garbage from, how they buy it, who they give the money, and how much is paid per skip. It was the researchers who compare this data with Mbarara municipality rules and procedures to establish what practices constituted corruption as discussed in the findings. Secondly, for other respondents who were aware of, but not necessarily participants in, corrupt practices and free to talk about them, they were asked to share what they think of corruption particularly as it relates to solid waste in the town, how it is perpetuated, the key actors, and possible remedies. Data on these different themes was collected using semistructured interview questionnaires/guides.

### 3.3. Date Collection Dates and Language Used

Data collection interviews were undertaken between 8^th^ and 20^th^ May 2019, by two researchers (the authors of this article) in the local language spoken in the area (Runyankole) and later transcribed in English. On average, each interview took between 30 minutes and an hour to execute.

### 3.4. Ethical Considerations

The entire research process was conducted with due respect to ethical considerations particularly keeping informants identity confidential. The researchers obtained informed consent of all respondents before their participation in the study. For those whose identities are openly identified, it was done with their informed consent. Primary data was triangulated with information obtained from secondary sources to come up with a nonnumerical analysis and article.

### 3.5. Presentations of Study Findings

Different corrupt actors perpetuate different forms of corruption which also affect solid waste management in different ways in Mbarara municipality as synthesized in Table 2 for easy understanding and explained later.

#### 3.5.1. Bureaucratic Corruption by Mbarara Municipal Officials

After the 2016 presidential elections, President Museveni promised the country that he was going to defeat public corruption which he mainly blamed on disoriented public officials. In December 2018, Museveni created a new Anticorruption Unit within State House headed by, Lt. Col. Edith Nakalema was mandated to investigate, expose, and fight corruption in the country. In January 2019, the Unit earnestly started its work and has so far investigated and exposed several public corruption scandals especially in district/municipal local governments. On February 24, 2019, acting on tip-offs from whistleblowers, the Unit conducted an impromptu investigation in Mbarara Municipality and arrested several officials over allegation of corruption. Those arrested included the Municipal Procurement Officer, the Municipal Physical Planner, the Internal Auditor, and the Municipal Engineer. The arrests were for corruption scandals related to illegal sale of public land, falsification of documents, inflating procurement costs, direct embezzlement of public procurement funds, and false accounting which all ran into hundreds of millions of shillings [36]. While the unearthed corruption scandals were not directly related to solid waste management it can be argued that it is such kind of money that if properly utilized would help fund some of the waste management operations such as procuring skips and repairing garbage trucks (interview, Chairman, Nyamitanga Division 11, May 2019). Two of the three very old garbage trucks owned by the Municipal Council broke down over six months ago and have remained unrepaired because of lack of money and, as a consequence, most of the garbage remains uncollected (interview, Principal Medical Officer of Health (PMOH) Mbarara Municipality, 9 May 2019; Rwebikoona Market Vendors leader, 10 May 2019).

#### 3.5.2. Corrupt Selling of Sorted Garbage

All the garbage (both compostable and noncompostable) collected in different parts of the municipality is supposed to be transported to the solid waste processing plant at Kenkombe in Kakoba Division [26]. The divisions' pay for the transportation costs (drivers and fuel), while the municipal council provides the trucks, covers the repair costs of trucks and pay workers at the dumping/processing site. At the processing plant, noncompostable solid waste such as scrap metal, plastic bottles, and other plastics are separated and later sold to dealers involved in this business. Noncompostable solid waste which may be of no further value is supposed to be put into the landfill. The compostable garbage is further processed into manure and sold to community users at Ushs 70,000 (US$ 18.6) (excluding transport) per ton. This money is supposed to be deposited on the municipal council bank account and is expected to cover most of the municipal solid waste management costs such as truck repairs, fuel, salaries for drivers, and garbage sorters (interview, Principal Medical Officer of Health (PMOH) Mbarara Municipality, 9 May 2019). However, the finding in this study revealed that very little compostable garbage reaches the solid waste dumping/processing plant at Kenkombe (interview, Site Manager, Kenkombe Solid Waste Dumping Site, 14 May 2019). Instead, it is usually sorted by a few informally hired people at the collection points (interview, Garbage Sorters on Rwebikoona Market, Kamukuzi Division, 17 May 2019) and directly sold by garbage truck drivers to farmers at different prices (a skip of sorted compostable garbage is usually bought at Ushs 40,000–60,000 including transport) (interviews with 10 farmers in Ngaara, Rwarire, and Kibingo villages in Katoojo Parish, Nyakayojo Subcounty (all these have bought this garbage manure from the drivers several times), 12 May 2019). The garbage truck drivers, their turn boys, garbage sorters, and some senior officials in the municipal Environment department share the money generated from this illegal sale of organic garbage (manure) (interviews, Key Informants, 13 May 2019) (Table 3).

**Figure 3:**
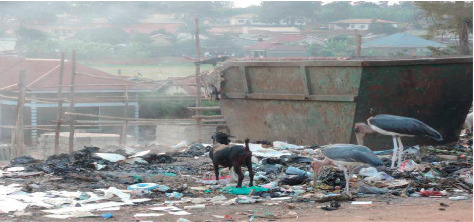
Uncollected garbage in Kakyeka area.

**Figure 4:**
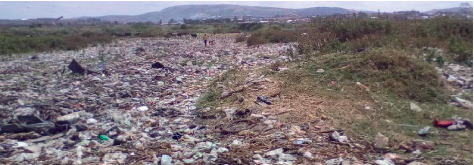
Uncollected garbage in Kenkombe waste dumping site.

As illustrated in Table 3, garbage truck drivers mainly focus on collecting and transporting this kind of garbage to people who can afford to pay them irrespective of the distance involved since the fuel and truck repairs are met by divisions and the municipal council (interviews, Key Informants, 13 May 2019). They also put most of their efforts in places such as markets which generate a lot of saleable/compostable garbage while residential areas and places whose garbage skips take a long time to fill take a very long time to have them collected resulting in littering (interviews, Key Informants, in Kakoba and Nyamitanga Division, 14 May 2019). This was evidenced by the researchers also reflected in the picture in Figure 3 taken in the Kakyeka area of Kamukuzi division:

Because the compostable solid waste is illegally and corruptly sold before reaching the processing plant, it is usually the nonuseful waste such as children used pampers, polythene bags, small pieces of charcoal, broken glasses, wood scrap from carpentry workshops, very low value metal scrap, and plastic bottles among others that find their way to the composting and landfill site (Int. Site Manager, Kenkombe; interview, Kakoba Division Council Official, 14 May 2019). This can hardly be turned into manure that can generate money expected to cover solid waste management running costs such as fuel, truck repairs, and salary for people working at the dumping/composting site (Int. Site Manager, Kenkombe; interview, Kakoba Division Council Official, 14 May 2019). As a consequence, workers at Kenkombe garbage dumping and processing site were laid off from December 2018 (interview, Key Informant, Mbarara Municipal Council, 9 May 2019). Continued dumping of solid waste at the site when workers are supposed to sort it or dump it in the landfill were laid off, resulting in a lot of open garbage decomposition and rotting, which comes with an unbearable stench and pollution of the environment around the dumping site (interviews, Four Household Heads in Rwemigyina and Kenkombe villages that border the Rwentondo (Kenkombe) garbage-dumping site in Kakoba, 15 May 2019) as exemplified by Figure 4.

#### 3.5.3. Inappropriate Disposal of Industrial Waste

**Figure 5:**
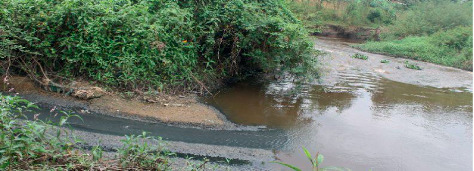
Untreated industrial waste flowing into River Rwizi.

According to the Mbarara Municipal Council Waste Management byelaws 2005, every factory operating within Mbarara Municipality is supposed to have a properly functioning treatment facility (kiln) to deal with its industrial waste. However, almost all the factories do not have these facilities and remain operational through bribing the municipal officials (interview, Key Informant, Mbarara Municipal Council, 17 May 2019). Many dump their industrial waste in unutilized public land, forests, or garbage skips meant for the general municipal solid waste (interview of 4 residents of Kateera Cell, Ruti Ward, Nyamitanga Division, 18 May 2019). Others transport their hazardous waste directly to the Kenkombe dumping site or just release it into river Rwizi (interview, Mbarara Municipality Principal Medical Officer of Health (PMOH), 9 May 2019; interview, Site Manager, Kenkombe), as evidenced in Figure 5.

The municipal leadership blames the problem of industrial waste on factory owners or managers who run their businesses without proper waste disposal facilities, which in itself is an abuse of entrusted authority (interview, Current Mayor (2016–Present), Mbarara Municipality, 16 May 2019). However, others placed this whole mess on Municipal Officials who take bribes from factory operators and turn a blind eye on them (interview, Former Mayor (2001–2016), Mbarara Municipality, 16 May 2019; residents of Kajoogo Cell, in Kamukuzi division, 16 May, 2019).

#### 3.5.4. Grabbing of the Dumping Site Land

The 114-acre Mbarara Municipal Council owned land in Kenkombe and Kakoba division serving as the municipal solid waste dumping site has not been spared by land grabbers. The seriousness of these land grabbers was brought to public attention in August 2018 when a Danish company (Transform AF) wanted to sign a memorandum of understanding with Mbarara Municipal Council to build an effective fertilizer making plant on this land. All of a sudden two individuals named Asiimwe Alex Mubangizi and Jacklet Mubangizi claimed ownership of the land in question. They made their claim in a letter submitted to the town clerk through the office of the Inspectorate of Government (IGG) in Mbarara and dragged the council to court [37]. As a result, the entire planned project to build an effective fertilizer making plant by the Danish company (Transform AF) has since been shelved as the company moved on and the land controversy is dragging on (interview, Principal Medical Officer of Health (PMOH), Mbarara Municipality, 9 May 2019). This is a very huge setback for Mbarara Municipality as it has for a long time expressed a desire to have a private operator that manages its waste (interview, Principal Medical Officer of Health (PMOH), Mbarara Municipality, 9 May 2019), a model that has been proven to be more effective in other places such Kampala [17]. Several high ranking commentators in the country including President Museveni, the *Katiikiro* (Prime Minister) of Buganda kingdom; Charles Peter Mayiga, the Minister for Lands; Hon. Betty Amongi, the Head of the High Court Land Division; and Hon. Justice Dr. Andrew Bashaija among others have all pointed at corruption as a big factor fuelling land grabbing which has reached epidemic proportions in the country [38–42].

### 3.6. Discussion of Study Findings

From the study finding presented above, it can be observed that both corruption and municipal solid waste management are huge and complex problem. There is a clear link between corruption and solid waste management challenges in Mbarara municipality. Seen from the broader context, this may not be surprising because corruption is now a systemic problem affecting almost all facets of public life in Uganda [43, 44]. While the findings that garbage truck drivers, their turn boys, and sorters who are directly supposed to help reduce garbage in the town turned the whole process into a lucrative business for themselves and a big problem for others may be a new revelation in the context of waste management particularly in Mbarara municipality, it is not new in the context of corruption. Studies in other cities have indeed indicated that people mandated to address a particular problem may simply use that opportunity to enrich themselves while making the whole problem worse. For instance, in cities such as Mumbai, Nairobi, and Lagos bribes extorted from street vendors by law enforcement officials sent to evict them from the streets run into millions of dollars per year [45, 46]. It is also clear that the wide spread corruption in Mbarara involves many actors who are working in coordinated ways. This is in agreement with other scholarly literature and reports which have stressed that, in societies where corruption is endemic, it is rarely an act of few individuals but syndicated groups [47–49]. The article also recognizes that while corruption is a key contributor to solid waste management challenges in Mbarara, it is not the only factor but it is reinforced or reinforces others including but not limited to poor financing, failure to enforce existing waste management laws, political interferences, lack of community participation, poor attitude by the public towards waste collection, inadequate planning, and people's tendency to throw garbage anywhere including environmentally sensitive areas (Table 4).

## 4. Recommendations

Addressing corruption and solid waste management challenges in Mbarara municipality requires targeted interventions that address issues raised by this paper in collaboration with those raised by other scholars/reports as discussed hereunder.

- Broadly speaking, the war on corruption in Uganda has mainly been lost due to impunity and selected punishment [51]. Yet in cities such as La Paz in Bolivia and Hong Kong in China and countries like Singapore or Rwanda where the war on corruption has been won, the trick was mainly severe punishment to anyone implicated in corruption without fear or favor [52–54]. It is therefore a key recommendation of this paper that anyone suspected of corruption should be properly investigated and anyone implicated should be severely punished without fear or favor. Anticorruption agencies and municipal administrators should widen their investigations always to include not only people directly by the municipal council but also other actors such surveyors that provide wrong information and mappings to land grabbers, land registry officials that fraudulently provide land grabbers with land titles, police officers that kill or provide wrong evidence, and judicial offices that give biased judgments among many others.
- All factories should be required to have appropriate solid waste management plans and facilities before they can be granted a license to operate in the municipality. Factories without proper facilities to manage their waste should have their operating licenses suspended until they put those facilities in place. The municipality should conduct regular and impromptu visits to these premises to make sure that those actors that may have those facilities are actually using them. Impromptu visits can sort this one out so that anyone found to be breaching the rules is fined accordingly.
- The courts of law should make cases such land grabbing a priority, expedite their hearings, and conclude them quickly. Punishments for people or companies proved by courts to have engaged in such land grabbing attempts that resulted in loss of huge public investments or cost the taxpayer a huge fortune should be given very severe penalties that can act as future deterrents to others whether land grabbers or corruption perpetrators (interview, Several Key Informants, 9–16 May 2019).
- Adoption of smart technologies which can help make city managers, waste management operators, and citizens active participants in the processes of reporting anomalous situations and easing business operation has helped improve solid waste management in cities like Milan and Stockholm, among others [2]. It is thus recommended that Mbarara municipality which is scheduled to become a city in July 2020 [55] embraces the use of smart technologies such as fitting garbage trucks with GPS that can track their movements which can then be collaborated with the information from the people who will have paid for manure, collection points, and the dumping sites. In cases where the trucks are observed to be moving outside the expected zones, then the drivers can be questioned (Key Informant, Mbarara Municipal Council).
- The method of requiring people who want to buy manure to first pick forms from the council, pay in the bank, and then go to the compost manure processing plant in Kenkombe is very costly in terms of both money and time. Municipal council officials should embrace new money transfer innovations such as mobile money payment techniques where farmers can just buy and pay for the compost they want using their phones any time which saves time. Then council can establish a coordinating office such that after payment, they are booked and their compost is delivered in the process (interviews with four farmers in Katoojo Parish, 11 May 2019).
- The government, CSOs, and municipal council should streamline and encourage practices that improve better settlement patterns, reduce slums, improve livelihoods of low income earners, and promote behavioral changes so as to discourage indiscriminate dumping of garbage [56].
- Almost all respondents interviewed for this study are fine with the idea of handing over the management of solid waste in Mbarara Municipality to a private investor/operator or a public private partnership on condition that such an understanding is entered into with clear terms that protect the public interest. Indeed, other studies in Uganda have demonstrated that these arrangements can work if well regulated [17]. Thus, this article recommends that the authorities in Mbarara Municipality carefully study this option and put in place measures to protect the public from any kind of abuse from private actors and if the appropriate actors are found, they should try out this option. This option cannot work alone unless the local population is collectively encouraged and attentively supervised to ensure that garbage is delivered at collection points when it is already sorted so as to reduce the burden of separating [2].

## 5. Conclusion

This research article has been able to expose and illustrate how corruption perpetuated by different actors and manifesting in different forms has made solid waste management in Mbarara Municipality a big challenge. The key conclusions of this research are that corruption in any form (whether political perpetuated by top politicians, bureaucratic by municipal officials, or petty by low ranking garbage truck drivers, turn boys, and sorters) can be very devastating by turning a simple and manageable challenge into a huge problem affecting an entire town. This research has also revealed that corruption can also be devastatingly perpetuated by private sector players such as clinics, hospital, and factory owners neglecting their responsibility in ways that may not be easily understood by lay people as corruption. It is the hope of the researchers that the revelations and knowledge generated from this research will be useful to anticorruption agencies and local authorities who are trying to address both corruption and solid waste mismanagement in Mbarara and other Ugandan Municipalities and beyond.

## Figures and Tables

**Table 1:** Sampling methods and selected key informants.

Category of respondents	Number interviewed
Municipal level politicians	3
Municipal level technocrats	2
Division level politicians	3
Respondents at cell level (one from each cell considered to have an acute solid waste management problem)	10
Official working at the Kenkombe solid waste treatment plant	1
Garbage truck drivers	1
Market vendor leaders	3
Garbage sorters	5
Farmers who have bought the garbage	9

**Table 2:** Corrupt actors, nature of corruption, and impact on waste management.

Corrupt actors	Nature of corruption	Impact on waste management
Driver, turn boys, and garbage sorters	Illegal sale of sorted garbage	Results in direct loss of revenue for the council as the money generated is taken by drivers, sorters, and turn boys and increased costs from truck breakdowns and fuel. Neglecting collection of garbage from which they can extract corrupt profits causes garbage overflowing in some areas

Bureaucrats	Illegal sale of public land, falsification of documents, inflating procurement costs, direct embezzlement of public procurement funds, false accounting	The hundreds of millions of lost public money could be used to buy garbage trucks and skips, hire more workers, pay for repairs, and even acquire more land in different divisions for dumping garbage. Most of these are not done because money and other resources are corruptly utilized

Land grabbers	Encroached and grabbed dumping site land	Foiled the much needed investment in waste management as investors could not build plant on contested land.

Politicians	Inadequate supervision of workers (drivers, turn boys, and sorters); inadequate monitoring of factory/hospital owners; refuse to enforce Mbarara municipality waste management byelaws for their own political reasons	Inadequate supervision of driver's means that they focus on collecting what is easy to sell and ignore the unprofitable garbage making some areas suffer from garbage. This has also allowed drivers to transport garbage to far distances costing council a lot of money in fuel and frequent truck break down. Inadequate supervision or taking of bribes from factory/hospital operators has resulted in indiscriminate dumping of deadly industrial and medical waste.

Factory/hospital owners	Dump their industrial/medical waste in public land, forests, or garbage skips meant for the general municipal solid waste	Pollute very sensitive areas such as rivers and swamps.

**Table 3:** Garbage sellers, prices, and beneficiaries or losers.

Garbage sellers	Prices	Beneficiaries or losers
Unauthorized garbage truck drivers and turn boys	Ushs 40,000–60,000 including transport	The beneficiaries of this illegal sale of manure/garbage are truck drivers, turn boys, garbage sorters, and farmers who get manure on cheaper prices. The loser is the taxpayer and the people whose garbage is uncollected.

Authorized managers at Kenkombe dumping site	Ushs 70,000 per ton excluding transport	The money from this sale goes to the municipal council bank accounts and is properly budgeted for. Particularly to pay for the operations of waste management at the dumping site. However, looking at the cost, it is more expensive and thus attracts fewer customers given that they can get it cheaply from garbage truck drivers. The losers in this process is the workers at the site who get less business, council loses revenue, and the public finally suffers with uncollected garbage

**Table 4:** Factors contributing to solid waste problems and key perpetrators.

Factors contributing to solid waste problems	Key perpetrators
Corruption	Municipal council technocrats, politicians, truck drivers, turn boys, factory/hospital owners, and farmers. This means that corruption is a complex problem involving different actors and manifests in different forms.

Poor financing	The central government is partly to be blamed. No money is allocated by the central government to cater for waste management. The council is supposed to use local revenues to finance this. However, their sources of revenue were crippled by the 2017 presidential directive that stopped collection of daily fees from market vendors and taxes drivers in the country [50]. In the specific case of Mbarara municipality, this was their key source of money (interview, Principal Medical Officer of Health (PMOH), Mbarara Municipality, 9 May 2019). Poor financing at the municipal level can be blamed on misprioritisation and also corruption

Failure to enforce existing waste management laws	Municipal political and bureaucratic officials and community members

Political interferences	Politicians both at the central government level (e.g., the presidential scrapping of collecting daily fees from market vendors and taxi owners) and at municipal, division, and village level.

Lack of community participation, poor attitude by the public towards waste collection and tendency to throw garbage anywhere including environmentally sensitive areas	All community members but particularly those in very low income areas

Inadequate planning	Central and municipal politicians and technical people

## Data Availability

The data (both primary and secondary) used to support the findings of this study are included within the article and cited accordingly.
